# Ectomesenchymal Chondromyxoid Tumour of the Dorsal Tongue Presenting with Impaired Speech

**DOI:** 10.1155/2016/7342910

**Published:** 2016-04-28

**Authors:** Laura A. Schep, Martin J. Bullock, S. Mark Taylor

**Affiliations:** ^1^Faculty of Medicine, Dalhousie University, 6299 South Street, Halifax, NS, Canada B3H 4R2; ^2^Division of Pathology, Dalhousie University, 6299 South Street, Halifax, NS, Canada B3H 4R2; ^3^Division of Otolaryngology, Head and Neck Surgery, Department of Surgery, Dalhousie University, 6299 South Street, Halifax, NS, Canada B3H 4R2

## Abstract

Ectomesenchymal chondromyxoid tumours (ECTs) are rare mesenchymal soft tissue neoplasms that typically present as a slow-growing asymptomatic mass on the anterior dorsum of the tongue. Our patient presented with impaired speech articulation and pain associated with upper respiratory tract infections when the lesion on his dorsal tongue would swell, and he would accidentally bite down on it. Microscopically, ECTs appear as unencapsulated, well-circumscribed proliferations of uniform round to fusiform cells embedded within chondromyxoid matrices. Most cases of ECT have been detected in the third to the sixth decades of life, with no sex preference. ECT may cause a range of symptoms that negatively impact patients' quality of life, including pain, dysphagia, odynophagia, bleeding, and, in the case of our patient, impairment of speech. We provide a unique preoperative clinical photograph and case description that should help readers in recognizing this neoplasm. Considering the rarity of ECT presenting clinically as well as in the literature, we believe this report will add to our growing understanding of ECT and its management. We report a case of ECT presenting on the anterior dorsal tongue that was successfully surgically resected under local anesthesia with clear margins, accompanied by a review of the pertinent literature.

## 1. Introduction

Ectomesenchymal chondromyxoid tumours (ECTs) are rare mesenchymal soft tissue neoplasms that typically present as a slow-growing asymptomatic mass on the anterior dorsum of the tongue, or, much less frequently, on the posterior tongue [1]. Microscopically, ECTs are identified as unencapsulated, well-circumscribed proliferations of uniform round to fusiform cells embedded within chondromyxoid matrices [1]. ECT has affected patients 7 to 78 years old and classically appears in the third through sixth decades of life, with literature review revealing no sex predilection. In the literature, ECTs have generally ranged in size from 0.6 to 0.7 cm to 1.2 to 2.0 cm in diameter [2], with very rare exceptions of much larger tumours reported [3]. We report a description of a 1.5 by 2.5 cm ECT presenting on the anterior dorsal tongue surface, accompanied by a dramatic preoperative photo. This ECT was successfully surgically resected under local anesthesia with clear margins. It is our hope that this case will help other clinicians recognize this important neoplasm and how to distinguish it from other entities that may share some histopathological commonalities.

## 2. Case Presentation

The patient is a 51-year-old male with a 15-year history of a slow-growing midline dorsal tongue lesion that was not causing any compressive symptoms. He had not experienced dysphagia or any impairment to his airway; however, the tongue lesion more recently resulted in a change in his speech articulation. He noted that there was only pain associated with upper respiratory tract infections, when the lesion would swell and he would occasionally bite down on it, resulting in mild bleeding. The patient was otherwise healthy. His previous history included diverticulitis in the descending colon, a hernia repair, colonoscopy, and chronic knee pain for which he was awaiting arthroscopy. His medications included ibuprofen and celecoxib and he had no known allergies.

**Figure 1:**
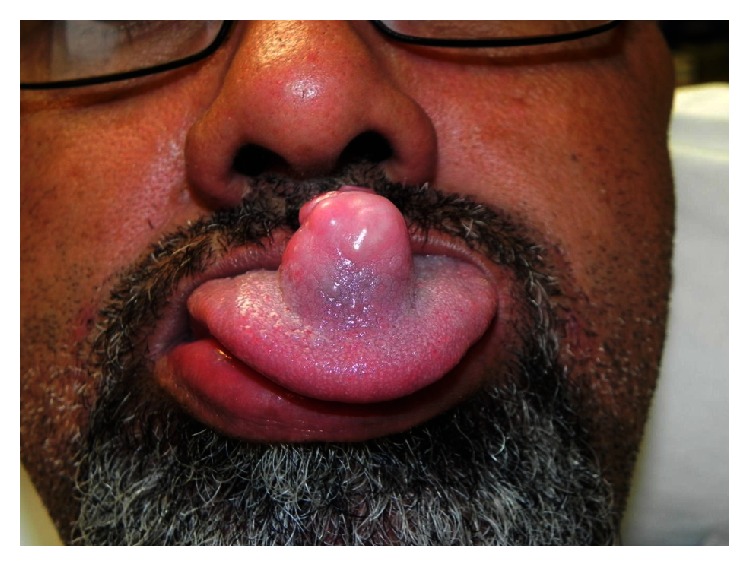
Preoperative photo demonstrating ectomesenchymal chondromyxoid tumour (ECT) of the dorsal tongue.

On oral examination, a 2.5 cm high × 1.5 cm wide dorsal tongue lesion with normal tongue papillae on the dorsal surface and mucosa on the undersurface was present (Figure 1). The mass was nodular and covered by intact mucosa. Small, healing ulcers were noted on the tip, which appeared to be traumatic. The base of the lesion was firm and it was nontender on palpation. No other mucosal lesions were seen in the oral cavity or oropharynx. The base of the tongue was soft on palpation, his larynx had a normal appearance, and nasopharyngoscopy was unremarkable.

The patient was offered excision for definitive management and to establish a definitive diagnosis. This was done under local anesthesia with 5 mm margins. A midline glossectomy was performed with dissection into the midportion of the deep substance of the tongue. The patient tolerated the procedure well and on follow-up the tongue had healed nicely with no evidence of recurrent disease. The final pathology was in keeping with an ectomesenchymal chondromyxoid tumour (ECT) with negative margins.

**Figure 2:**
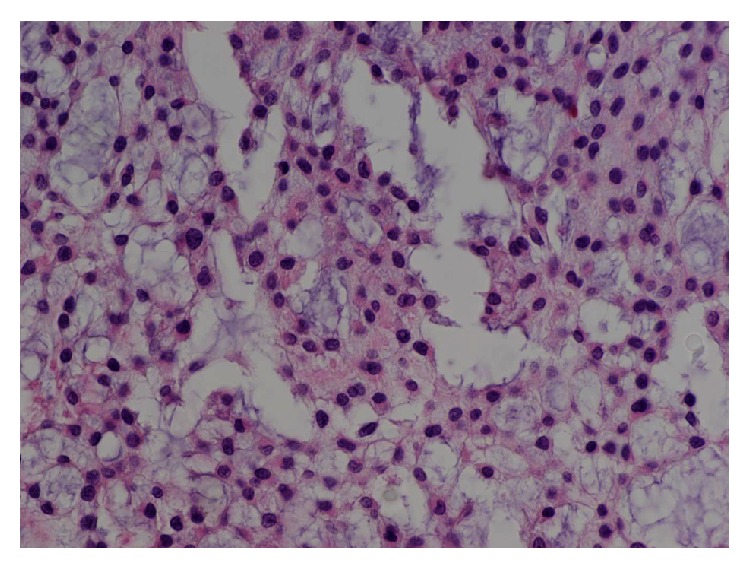
High-power image of tumour showing reticular pattern of uniform polygonal cells. From this photograph one can appreciate the ill-defined polygonal cell borders, on a background of pale grey-blue “myxoid” stroma (H&E stain, 400x).

Microscopically, the tumour was lobulated but generally well-circumscribed, extending from just beneath the surface epithelium into the underlying skeletal muscle. It consisted of ovoid to spindle-shaped cells with ill-defined cell borders and uniform nuclei (Figure 2). The cells were organized in a reticular (net-like) and focally microcystic growth pattern, with accumulation of myxoid and focally chondromyxoid stroma between cells. No worrisome features such as nuclear atypia, mitotic activity, or necrosis were identified. Immunohistochemistry was performed, which showed that the tumour cells were diffusely positive for S100 and glial fibrillary acidic protein (GFAP). Immunohistochemistry also revealed focal positivity of variable intensity and extent for keratin AE1/AE3, neuron specific enolase, smooth muscle myosin heavy chain, muscle specific actin, and desmin. Immunohistochemistry was negative for epithelial membrane antigen, CK8/18, CK5/6, CD34, and p63.

## 3. Discussion

ECT is a rare and benign neoplasm of the oral cavity, classified by the World Health Organization (WHO) in the pathologic spectrum of soft tissue myoepithelioma. The entity was first described by Smith et al. in 1995 [4]. In this initial manuscript, the authors presented a total of 19 ECTs, all located on the anterior surface of the tongue, that did not fulfill the diagnostic criteria for any other intraoral soft tissue chondromyxoid lesion at the time, while all have similar unique immunohistochemical and clinicopathological features. The authors proposed that the descriptive name “ectomesenchymal chondromyxoid tumour” be adopted until the histogenesis of this lesion became clearer. Over two decades later, this original name persists, as does the rarity of this lesion, with approximately 55 cases currently reported in the English literature. However, there have been some theories that have attempted to explain the histogenesis of ECT, which will be discussed here.

Similarly to Smith et al.'s original findings, the vast majority of subsequently described ECTs have been documented on the anterior tongue surface, although there are a handful of cases in which tumours have been located on the posterior aspect of the tongue [1, 5] and one case where the neoplasm grew on the hard palate of a 13-year-old boy, described by Gouvêa et al. in 2012 [6]. There was another reported case of ECT on the hard palate reported in 2006; however, the diagnosis in this case has been the subject of controversy due to an apparent lack of appropriate documentation [7].

In Smith et al.'s original report, the ECTs had been documented as occurring in men and women aged 9 to 78 years, with a median age of 32 years. The tumour's presence and growth ranged in duration from a few months to 10 years, and tumour size ranged from 0.3 cm to 2.0 cm [4]. A more recent review of 7 cases of ECT in 2015 reported an age range of 7 to 57 years, with a median age of 45.8 years, and the duration of the tumours was reported as “only available on two cases and ranged from six months to several years,” with sizes that ranged from 0.6 × 0.7 cm to 1.2 × 2.0 cm [2]. It is likely difficult to gauge the duration of this neoplasm considering its nonpainful, asymptomatic nature and tendency to grow slowly. Of note, in 2014, a case of ECT was published in the Dentistry literature showing a striking photo of the largest documented case of ECT, presenting in a young child of only 7 years; this tumour measured 5.0 cm × 3.0 cm × 3.0 cm and again was located on the anterior, dorsal aspect of the tongue [3]. In our patient, the mass was noted to have been present for 15 years. His ECT appeared on the anterior aspect of the tongue as per the majority of these lesions and in the time of life we would expect. The size of the lesion on his tongue (1.5 cm wide by 2.5 cm high) was larger than most documented ECTs, and its presence of 15 years was longer than the duration of most of these tumours in the literature as well.

### 3.1. Immunohistochemistry

Along with their original clinical review of ECT in 1995, to help with diagnosis, Smith et al. conducted thorough immunohistochemistry on the 19 cases presented. Results of this demonstrated strong positivity for glial fibrillary acidic protein (GFAP) in nearly all cases (18 of 19) and variable reactivity to CD-57/Leu-7 (8 of 9 cases were positive), occasionally positive for S100 (with these ranging from faintly to intensely positive), nonreactive to intensely reactive for cytokeratins AE1/AE3, and negative for epithelial membrane antigen (EMA) and desmin [4]. In the subsequent literature on ECT, many researchers have used a similar profile of immunohistochemistry for diagnosis of ECT, with a varying degree of expression and reactivity to these markers.

The immunohistochemistry performed for our patient's ECT showed diffuse positivity for S100 and glial fibrillary acidic protein (GFAP), focal positivity (of variable intensity and extent) for keratin AE1/AE3, neuron specific enolase, smooth muscle myosin heavy chain, and muscle specific actin and desmin. Immunohistochemistry was negative for EMA, CK8/18, CK5/6, CD34, and p63. The presence of staining for markers of both epithelial (AE1/AE3) and muscle differentiation (myosin, actin, and desmin) supports the concept that ECT is related to soft tissue myoepithelioma.

A recent case by Shogo and Koda from 2015 described the potential usefulness of CD56 as an adjunct marker for diagnosis of ECT, arguing that its inclusion may result in more frequent encounters and resulting diagnoses of ECT [8]. Historically, authors have commonly tended to use CD57, which is suggestive of neurogenic lineage, in the immunohistochemistry of ECT. This follows from Smith et al.'s original inclusion of this marker in their introductory report on ECT [4]. Indeed, according to the recent review by Aldojain et al. and the compilation of several other cases by Shogo and Koda, it is reported that 75–80% of cases of ECT show positivity for CD57 [2, 8]. Shogo and Koda argued that CD56 would be a useful adjunct in diagnosing ECT [8], considering it has been shown to be a more useful marker of neurogenic lineage compared to CD57 [9] and also tends to be more readily available in pathology laboratories. The result of CD56 immunostaining is not available for our case, though this may be a useful avenue for future research.

### 3.2. Differential Diagnosis

Diagnosis of ECT takes into consideration the clinical, light microscopic, and immunohistochemical findings. In particular, the clinical, immunohistochemical, and histopathologic features of ECT still remain to be definitively established, in large part due to the scarcity of ECT in the literature. However, this lack of definition of ECT's features likely also results from it being confused with or mistaken for other entities that share some of its histopathological features. Entities to include in the differential diagnosis include myoepithelioma, nerve sheath myxoma, mucocele, pleomorphic adenoma, oral focal mucinosis, glial choristoma, ossifying fibromyxoid tumour, chondroid choristoma, soft tissue myxoma, and cellular neurothekeoma [2, 10, 11]. The development and refinement of immunohistochemistry studies, and the identification of specific molecular markers, have allowed pathologists to distinguish ECT from other entities in the differential diagnosis. The majority of the above tumours are keratin negative. In our case, the major differential diagnosis was ossifying fibromyxoid tumour, but it was considered less likely because of the location, lack of ossification, and the keratin positivity, which is uncommon in these tumours. The lack of chondromyxoid matrix or a distinct luminal or glandular component, as well as the location, is also inconsistent with pleomorphic adenoma.

### 3.3. Histogenesis

Smith et al. originally offered several hypotheses in 1995 regarding the histogenesis of ECT, which is still a subject of dispute and remains to be established. Considering the tumour's nearly exclusive location on the dorsal anterior segment of the tongue, which is known to be derived from the first branchial arch during embryogenesis, it is widely held that ECTs arise from uncommitted ectomesenchymal cells that migrated from the neural crest. This was one of Smith et al.'s hypotheses and remains widely held at this point in time, although theories of muscle cell and myoepithelial cell origins have also been considered [4, 12, 13]. As the anterior two-thirds of the tongue are generally lacking in minor salivary glands, this theory would necessitate the tumours being “soft tissue myoepitheliomas” rather than of salivary origin.

Interestingly, a group of authors (as yet unpublished) have recently demonstrated EWSR1 rearrangement in a subset of 2 of 9 tumours [14]. There was no correlation between the translocation-positive ECTs and other molecular features of the tumours. These authors argue that the presence of the EWSR1 rearrangement in a fraction of ECT is evidence to support the theory that at least a portion of ECTs, from a cytogenetic perspective, are related to soft tissue myoepithelial neoplasms [14]. Evidently, more studies are needed to definitively elucidate the histogenesis and genetic profile of ECT.

### 3.4. Treatment and Recurrence

ECT is a benign entity, though it can impact important functions such as swallowing and speech, thus affecting patients' quality of life. Surgical excision remains the mainstay of treatment. Smith et al., in their original article, made note of rare recurrences of ECT following excision; in one case, the tumour was reexcised with repeat surgery and did not subsequently reappear, while follow-up information regarding the other recurrent ECT was not provided [4]. More recently, Portnof et al. reported a case that they considered a recurrence of ECT 5 years following initial presentation, after previously being misdiagnosed as a nerve sheath myxoma and then a low-grade sarcoma before the final diagnosis of ECT was established [12]. Although it is possible that this was a new lesion and not a recurrence several years after initial excision, this interesting case highlights the ongoing need for more studies and for better dissemination of knowledge of how to identify ECT, so as to prevent misdiagnoses. We are hopeful that our case will aid in this process. Our patient has been followed up consistently in the Outpatient Otolaryngology Clinic for over a year since his surgery and has not experienced any recurrence of ECT.

## 4. Conclusions

Ectomesenchymal chondromyxoid tumour (ECT) is an uncommon, benign tumour of the oral cavity that typically presents on the dorsum of the anterior tongue. The recommended treatment is conservative surgical removal. Further studies will need to be carried out to better delineate the histologic and genetic origins of this interesting and rare neoplasm.

## References

[1] Cardin M.-J., Fiset P. O., Zeitouni A. G., Caglar D. (2014). Ectomesenchymal chondromyxoid tumour of the posterior tongue. *Head and Neck Pathology*.

[2] Aldojain A., Jaradat J., Summersgill K., Bilodeau E. A. (2014). Ectomesenchymal chondromyxoid tumor: a series of seven cases and review of the literature. *Head and Neck Pathology*.

[3] Kale H., Mistry D., Vasant R., Jadeja N., Baranwal M. (2014). Ectomesenchymal chondromyxoid tumor: a rare case report. *Contemporary Clinical Dentistry*.

[4] Smith B. C., Ellis G. L., Meis-Kindblom J. M., Williams S. B. (1995). Ectomesenchymal chondromyxoid tumor of the anterior tongue: nineteen cases of a new clinicopathologic entity. *The American Journal of Surgical Pathology*.

[5] Seo S. H., Shin D. H., Kang H. J. (2010). Reticulated myxoid tumor of the tongue: 2 cases supporting an expanded clinical and immunophenotypic spectrum of ectomesenchymal chondromyxoid tumor of the tongue. *American Journal of Dermatopathology*.

[6] Gouvêa A. F., Díaz K. P., Léon J. E., Vargas P. A., de Almeida O. P., Lopes M. A. (2012). Nodular lesion in the anterior hard palate. *Oral Surgery, Oral Medicine, Oral Pathology and Oral Radiology*.

[7] Nigam S., Dhingra K. K., Gulati A. (2006). Ectomesenchymal chondromyxoid tumor of the hard palate—a case report. *Journal of Oral Pathology and Medicine*.

[8] Shogo T., Koda K. (2015). A case of a CD56-expressing ectomesenchymal chondromyxoid tumor of the tongue: potential diagnostic usefulness of commonly available CD56 over CD57. *International Journal of Clinical and Experimental Pathology*.

[9] Mechtersheimer G., Staudter M., Möller P. (1991). Expression of the natural killer cell-associated antigens CD56 and CD57 in human neural and striated muscle cells and in their tumors. *Cancer Research*.

[10] Allen C. M. (2008). The ectomesenchymal chondromyxoid tumor: a review. *Oral Diseases*.

[11] Leeky M., Narayan T. V., Shenoy S., Jamadar S. (2011). Ectomesenchymal chondromyxoid tumor: review of literature and a report of a rare case. *Journal of Oral and Maxillofacial Pathology*.

[12] Portnof J. E., Friedman J. M., Reich R., Freedman P. D., Behrman D. A. (2009). Oral ectomesenchymal chondromyxoid tumor: case report and literature review. *Oral Surgery, Oral Medicine, Oral Pathology, Oral Radiology and Endodontology*.

[13] Woo V. L., Angiero F., Fantasia J. E. (2005). Oral myoepithelioma of the tongue. *Oral Surgery, Oral Medicine, Oral Pathology, Oral Radiology, and Endodontology*.

[14] Argyris P., Bilodeau E., Trochesset D. A subset of ectomesenchymal chondromyxoid tumors (ECTs) of the tongue shows EWSR1 rearrangement and is genetically linked to soft tissue myoepithelial neoplasms: a study of 9 cases.

